# The Dynamic Interaction between Extracellular Matrix Remodeling and Breast Tumor Progression

**DOI:** 10.3390/cells10051046

**Published:** 2021-04-29

**Authors:** Jorge Martinez, Patricio C. Smith

**Affiliations:** 1 Cell Biology Laboratory, INTA, University of Chile, Santiago 7810000, Chile; 2 School of Dentistry, Faculty of Medicine, Pontificia Universidad Católica de Chile, Santiago 8330024, Chile

**Keywords:** collagen remodeling, desmoplasia, breast cancer

## Abstract

Desmoplastic tumors correspond to a unique tissue structure characterized by the abnormal deposition of extracellular matrix. Breast tumors are a typical example of this type of lesion, a property that allows its palpation and early detection. Fibrillar type I collagen is a major component of tumor desmoplasia and its accumulation is causally linked to tumor cell survival and metastasis. For many years, the desmoplastic phenomenon was considered to be a reaction and response of the host tissue against tumor cells and, accordingly, designated as “desmoplastic reaction”. This notion has been challenged in the last decades when desmoplastic tissue was detected in breast tissue in the absence of tumor. This finding suggests that desmoplasia is a preexisting condition that stimulates the development of a malignant phenotype. With this perspective, in the present review, we analyze the role of extracellular matrix remodeling in the development of the desmoplastic response. Importantly, during the discussion, we also analyze the impact of obesity and cell metabolism as critical drivers of tissue remodeling during the development of desmoplasia. New knowledge derived from the dynamic remodeling of the extracellular matrix may lead to novel targets of interest for early diagnosis or therapy in the context of breast tumors.

## 1. Tumoral Extracellular Matrix

Breast cancer corresponds to the most frequently diagnosed malignancy, affecting 2.3 million new cases each year [1]. A striking feature of breast tumors is the development of a desmoplastic tissue component that is actively synthesized and remodeled by stromal cell populations [2]. This specific compartment corresponds to a dense connective tissue characterized by the abundance of fibrillar collagens that includes types I and III collagen along with increased proteolysis of type IV collagen [3]. This dense structure allows the early identification of tumors and also represents a critical factor for tumor development and progression [4].

High heterogeneity has been described in the desmoplastic tissue, ranging from a mostly cellular stroma (that comprises fibroblasts, endothelial and immune cells with little extracellular matrix (ECM)) to a dense structure with few cells and a high proportion of fibrillar collagens [3,5]. In desmoplastic mammary tissue, tumor stroma also shows a reduction in adipocytes size, the most abundant tissue component of normal breast structure [6,7]. Originally, desmoplasia was interpreted as a reaction of the host against tumor cells. Therefore, it was entitled as “desmoplastic response” [8]. Interestingly, mammographic studies were able to detect desmoplastic tissue in normal human breasts identified as areas of dense tissue when compared to the predominantly mammary fat tissue. Therefore, it was designated as “mammary density” (MD). Accordingly, radiodense areas are characterized by several histologic features of malignant stroma including a reduced proportion of adipocytes and increased presence of stromal cells and fibrillar collagens [9]. Conversely, the finding that desmoplastic tissue has been identified in the absence of tumor cells suggests that desmoplasia may not correspond to a response to invasive malignant cells, but is a prior condition aiding the development of cancer [10]. In fact, MD, understood as the result of the increased proportion of collagens relative to fat-tissue content, has arisen as one of the most relevant non-genetic markers of breast cancer risk.

An involvement of 60% or more of the breast tissue with mammographically dense structure confers a three-to-fivefold increase in relative risk for breast cancer [11]. Type I collagen is the major component of the stromal ECM network that determines mammary density [12]. Collagen structure and crosslinking acts as a scaffold allowing cancer cells to migrate and invade surrounding tissue and is thus associated with metastasis and poor prognosis in breast cancer patients [12]. Besides collagen, other extracellular matrix components such as hyaluronic acid are also important components of most solid tumors and their coordinate accumulation is often associated with the creation of a more robust stroma for tumor growth and a worsened prognosis [13]. Tumor accumulation of these matrix components is associated with an increased tumor interstitial pressure, the collapse of tumor vasculature, and the consequent development of a hypoxic phenotype [14]. Some leucine-rich proteoglycans, such as Lumican and Decorin, also display a higher expression in high density versus low density tissue and are directly associated with higher tumor grade [15]. 

Collagen-dependent tumor rigidity exerts a crucial influence in epithelial behavior. Interestingly, it has been demonstrated that collagen fibril diameter—and not pore size—is a primary determinant that regulates cell morphology, cluster formation, and invasion in a breast cancer model [16]. Tumor rigidity is not the same in its whole tumor tridimensional structure. Using the MMTV-PyMT transgenic mice, a standard animal model of human breast cancer, it was possible to detect by atomic force microscopy (AFM) that the periphery of the tumor exhibits ≈7 times more stiffness when compared to the interior, confirming previous data showing that human breast tumor biopsies possessed a collagen-rich invasive front that is characterized by increased mechanical stiffness over the highly cellular and ECM-deficient necrotic core [17]. In addition to this, the heterogeneity of tumor microarchitecture and its role in cell migration was also demonstrated in an in vitro 3D model. In this study, highly metastatic MDA-MB−231 breast adenocarcinoma cells, tend to migrate from the dense collagen matrix to the loose area suggesting that these cells showed a tendency to migrate towards a more compliant matrix [18].

In parallel, after tumor initiation, many of the anisotropic collagen fibers progressively thicken and linearize, allowing tumor cells to migrate along this “highway” of collagen fibers oriented radially away from the tumor boundary. Therefore, rigidity expressed in the cellular invasive front and in the extracellular space could enhance cell migration, a key step in tumoral progression [19]. In human breast tumors, the establishment of this stiffer phenotype during the carcinogenic development increases their rigidity (from 0.8 to 4.0 kPascals), which activates force-dependent signaling routes that lead to sustained changes in cell behavior associated with poor prognosis [20].

Cancer progression is frequently associated with a chronic inflammatory condition that results in the migration of different cell populations into tumor microenvironments [21]. In the case of breast cancer, it has been described that macrophages are one of the more abundant infiltrating cells that conform, in association with adipocytes, crown-like structures [22]. Experiments performed using three-dimensional (3D) collagen matrices with different fibril density showed that the increase of matrix density modulates macrophage polarization, enhancing pro-inflammatory milieu by M1 macrophages [23]. These results confirm previous data showing that THP−1 derived macrophages cultured on collagen-coated polyacrylamide gels of varying stiffness adapt their polarization state, functional roles, and migration capacity according to the stiffness of the substrate. Stiff polyacrylamide gels prime macrophages towards a pro-inflammatory phenotype while soft gels prime cells towards an anti-inflammatory, highly phagocytic phenotype [24]. Additionally, using 3D cultures, it has also been demonstrated that T cell proliferation was significantly reduced in a high-density collagen-derived matrix compared to a low-density counterpart, conditions that induce a reduction in the number of infiltrating CD8+ T-cells, but do not affect the proliferation of breast cancer cells [25].

Remodeling of the ECM under physiological and pathological conditions convoke a variety of active molecules. One of the most intriguing is SPARC (secreted protein acidic and rich in cysteine, also termed osteonectin), a glycoprotein that belongs to the matricellular group of proteins [26]. SPARC is highly expressed in the tumor stroma, principally in peritumoral fibroblasts, and its expression (mainly associated with collagen and vitronectin) has been associated with poorer prognosis [27]. Breast cancer is not an exception of these situations, as was described by Helleman et al.; who reported that SPARC expression levels were significantly associated with a shorter metastasis-free survival [28]. However, there is also evidence that overexpression of SPARC in the stroma of breast cancer is associated with a decreased risk of bone metastasis, the main site of metastases for this type of tumor [29]. This paradoxical result probably reflects the bone-forming activity of SPARC that enhances the survival of osteoblasts and stromal cells [30]. Nevertheless, the role of SPARC in tumor ECM is still controversial and is probably explained by the wide range of functions attributed to this particular protein.

MMPs (metalloproteinases) are active agents of matrix turnover. They represent a large group of at least 26 calcium-dependent and zinc-containing endopeptidases, which have been implicated in the remodeling and degradation of different components of the ECM, which includes elastin, gelatin, fibrillar collagens, and proteoglycans [31]. MMPs are frequently expressed at minimal levels in resting normal tissues. However, their expression is frequently stimulated in conditions like wound healing, tissue development, inflammatory conditions, and cancer [32]. MMPs are regulated by hormones, growth factors, and cytokines, and are involved in ovarian functions [32]. Endogenous MMP inhibitors and tissue inhibitors of MMPs (TIMPs) strictly control these enzymes [33]. An example of the role exerted by MMPs in breast cancer is the MMP-9-dependent enhancement of breast cancer malignancy through activation of the TGF-β/SMAD signaling [34]. MMP-14 (a membrane-bound enzyme) is another important molecular component that allows the acquisition of a malignant phenotype by the proteolytic activation of soluble MMP-2 and -13, providing a clearer path for cancer cells to migrate by the proteolysis of a variety of ECM components (e.g.; gelatin, fibronectin, collagen) at pericellular sites, among other functions [35]. MMP-14 is also a crucial protagonist in malignant-promoters processes. For example, MMP-14 empowers breast cancer TICs (tumor-initiating cells or cancer stem cells) to initiate tumors and activate motile programs under hypoxic nutrient-deprived conditions [36]. Given the function of MMPs in matrix remodeling, a fundamental process in tumor development and metastasis, future studies will probably reveal important roles for these enzymes in breast cancer.

## 2. Obesity and Its Impact on Desmoplastic Tissue Remodeling 

In spite of the fact that obesity has been associated with higher cancer mortality for decades, there is still limited knowledge regarding the mechanisms involving obesity and its impact on desmoplastic matrix remodeling [37]. Studies performed in mouse models of obesity (diet and genetically induced) demonstrated that an increased number of myofibroblasts in mammary adipose tissue generates a more fibrillar and stiffer ECM, abundant in type I collagen and fibronectin. This condition worsens desmoplasia and related ECM alterations in mammary tumors [38]. The same group of researchers recently demonstrated that fibrillar collagen networks are able to mechanically regulate myofibroblastic differentiation of adipose-derived stem cells (ASCs) favoring in this manner fibrosis. They described that collagen networks with thicker fibers and larger pores exhibit a higher capacity to differentiate ASCs into α-smooth muscle actin positive, contractile, and proangiogenic myofibroblasts [39].

An ECM component that has attracted attention because of its association with obesity is collagen VI, a molecule that has been found up-regulated in both obese and tumor ECM. In a murine model, it was proposed that collagen VI released Endotrophin (ETP) as a product of processing, a phenomenon that is favored in the context of obesity. ETP participates in the fibrotic remodeling of breast ECM and also constitutes a critical mediator of tumor progression in obesity. Part of this stimulus is accomplished by stimulating TGF-β–dependent epithelia-mesenchymal transition (EMT) in the context of mammary tumors to potentiate pro-metastatic effects [40]. In a more recent work, it was demonstrated that collagen VI promotes cell adhesion, 2D migration, and 3D invasion in triple-negative breast cancer cells (TNBC) by signaling through the proteoglycan NG2 and β1 integrins, both of which can cross-talk with receptor tyrosine kinases that further activate the epidermal growth factor/mitogen activated protein kinase signaling axis [41]. 

Myofibroblasts are major regulators of fibrotic and desmoplastic remodeling and, therefore, the study of their origin and expansion represents a key issue to unravel desmoplastic development and their tumoral consequences [42]. Probably the best characterized markers for mammary density is CD36, a transmembrane receptor involved in adipocyte differentiation (among other functions) that is expressed in significantly low levels in high-density associated fibroblasts compared with their low-density associated counterpart in the breast tissue of disease-free women. Interestingly, downregulation of CD36 gene expression is also observed in carcinoma associated fibroblasts (CAFs) compared to fibroblasts derived from healthy tissue. It has been suggested that its low expression can be considered an early event in tumor formation [43].

ECM stiffness is also involved in cancer progression by modulating intrinsic properties of cancer cells. Indeed, a rigid ECM can cause intracellular contractions that provoke changes in actin cytoskeleton that favor cell migration [44]. Moreover, ECM stiffness is responsible for the activation of transforming growth factor-β (TGF-β) signaling that mediates epithelial-to-mesenchymal transition in cancer cells. Together, a complex interaction between epithelial and stromal cells plays a determinant role in tumor progression. In this association, tumor-associated fibroblasts are able to produce a tumor-supportive microenvironment that can promote the expansion of a pre-neoplastic lesion, but also opens the possibility of therapeutic interventions.

## 3. Tumoral Stiffness and Response to Therapy

It has been demonstrated that the existence of a stiffened tumoral microenvironment affects the sensitivity of cancer cells to chemotherapeutic treatment [45]. Different hypotheses have been proposed for this phenomenon, the most common being the difficulty of vascular morphogenesis to progress into a stiffer structure of tumoral microenvironment [46]. The stiffer intratumoral organization, composed mainly by fibrillar collagen, would generate an increased interstitial fluid pressure that disrupts drug delivery [47]. The specific participation of collagen as a dense structure that impedes drug delivery has been tested in experiments where collagenase treatment enhances drug delivery even for large molecular weight agents such as antibodies [48]. 

The use of three dimensional (3D) in vitro tumor models that can replicate the tridimensional microarchitecture of a tumor has made it possible to enhance drug development for cancer therapy and to improve the predictability of toxicity and drug sensitivity in cancer [49]. Using this technology, it has been shown that highly crosslinked collagen forms a physical diffusion barrier for small molecules that effectively protects tumor cells from drugs and, potentially, protects malignant cells from exposure to therapeutics [50]. Authors identified lysyl oxidases (LOX), as the agents responsible for the reduced diffusion through the ECM. By analyzing microarray datasets of pretreatment human biopsies from ovarian, colon, and breast carcinoma patient groups, with available follow-up information on response to chemotherapy, authors identified that chemoresistant tumors appeared to have higher expression of ECM-related genes, finding collagens I–V upregulated in the resistant tumors. Together, the lysyl oxidases LOXL1 and LOXL2 genes were also significantly elevated in the same group of resistant tumors [50]. Thus, a tumoral microenvironment consisting of an abundant fibrillar collagen mass and a high degree of cross-linking disfavor drug transport. 

Human epidermal growth factor receptor 2 (HER2)-positive breast cancers are very aggressive tumors that display a poor response to hormone treatment and are treated more successfully with tyrosine kinase inhibitors such as lapatinib [51]. It has been demonstrated that the efficacy of lapatinib to inhibit HER2-amplified breast cancer cells is severely reduced in a rigid microenvironment in a phenomenon that depends directly on the YAP and TAZ Hippo pathway transcriptional coactivators [52]. 

Resistance to water soluble molecules is frequently attributable to glycosaminoglycans (GAGS), hydrophilic molecules that establish an ionic interference with this transport [53]. In contrast, the transport of larger molecules, as antibodies, is affected mainly by total tissue content of collagen that, in cooperation with GAG components, creates a barrier to macromolecule motion in tumoral tissues [54]. Additionally, levels of hyaluronan (HA), a major extracellular matrix (ECM) glycosaminoglycan, have been correlated with poor clinical outcomes in several malignancies, including breast cancer. However, the influence of HA in tumor cells seems to depend on its molecular weight. It has been demonstrated that the inhibitory activity of HA in cellular invasion and the expression of epithelial markers (that denote a more benign phenotype) is an attribute of the high molecular weight species [55].

A difficult vasculogenesis provokes not only a complex drug delivery to tumor structure, but also the establishment of a hypoxic environment due to the diminished oxygenated blood supply. The relationship between hypoxia and ECM-dependent cancer progression is not straightforward. On the one hand, it has been established that hypoxia, by stabilization of the transcription factors hypoxia-inducible factors (HIFs), induces the expression of members of the lysyl oxidase (LOX) family, enzymes that catalyze the cross-linking of collagen molecules, which once secreted into the ECM are able to promote breast cancer metastasis [56]. On the other hand, hypoxia can also up-regulate numerous proangiogenic factors, including vascular endothelial growth factor and MMPs that constitute a powerful tool to counteract hypoxia [57]. Moreover, under hypoxic conditions, interstitial collagenases, such as MMP−1 that is able to degrade extracellular fibrillar collagen, generates not only a compliant environment that allows drug delivery but also provides metastatic tumor cells the ability to extravasate from the primary tumor tissue into the blood and lymph to colonize distant metastatic sites [58]. However, fibrosis and angiogenesis can arise simultaneously as occurs in pancreatic cancer models where pancreatic stellate cells (PSCs) isolated from human tumoral specimens and subjected to a hypoxia environment generate both a profibrogenic and a proangiogenic response [59]. Therefore, the role of hypoxia on the establishment of a stiffer microenvironment that promotes cancer progression is a multistep molecular process whose final outcome depends on the balance of apparently contradictory processes. 

## 4. A Matter of Signaling

Cells that constitute the tumoral microenvironment are able to convert the extracellular mechanical cues into intracellular biochemical signals. To do so, cells express a repertoire of membrane receptors to accomplish this function. Integrins are one of the most active receptors to collagens. Native collagen is recognized by four integrin heterodimers: α1β1, α2β1, α10β1 and α11β1. Integrin α1β1 binds both types I and IV collagens whereas α2β1 only binds type I collagen [60]. Another family of receptors that is positively associated with cell adhesion to collagen are the discoidin domain receptors (DDRs), a tyrosine kinase receptor family that plays a relevant role in the fibrotic phenomenon in a variety of systems [61]. In the case of integrins, upon binding to ECM molecules (such as collagen), they become allosterically active and, in this conformation, form adhesion clusters, which in cultured cells are denoted as focal adhesions (FA). This process involves the autophosphorylation of focal adhesion kinase (FAK) and the activation of a mechanosensitive cascade that includes an array of proteins, such as RhoA, Talin and vinculin, which provide a physical link between the integrins and actin cytoskeleton [62]. It has been found that exposure to a “stiff” two-dimensional substrate promotes FA size and strength [63], and that higher matrix stiffness results in increased adhesion signaling and chronically elevated activation of a FA-Rho GTPase-MAPK network that may explain part of the mechanism behind increased epithelial proliferation and cancer risk in human patients with high breast tissue density [64]. The same group has shown that FAK (a central piece of this complex) was found over-expressed in human breast carcinomas [65]. 

DDRs also play a role in cancer progression by regulating the interactions of tumor cells with the collagen matrix. The more specific trait of DDRs (1 and 2) is their tyrosine kinase activity that makes them part of the group of tyrosine kinase receptors (RTK) with the difference that, in this case, the receptor is activated by native collagen. This converts DDR in mediators of the cross-talk between tumor cells and their immediate collagenous matrix. Unlike conventional RTKs, their DDRs-stimulated signal is delayed and persistent. Therefore, the DDRs behave as part of a signaling network that translates continued information from the ECM acting as the key regulators of cell-matrix interactions [66]. DDR1 and DDR2 display a broad collagen specificity with fibrillar collagens acting as ligands for both receptors. However, DDR1, but not DDR2, also binds to collagen IV [67]. 

DDRs have been linked to tumor progression in several human cancers where their expression or activation is probably deregulated. However, their specific role in the progression of disease is still a matter of controversy, given that DDRs can also act as an anti-tumorigenic factor, depending on the type of cancer and ECM characteristics [68]. In specific types of cancer, when examining a large cohort of melanoma tissues by immunohistochemical staining it has been recently demonstrated that DDR1 expression was significantly associated with poor patient survival [69]. Epithelial-mesenchymal transition (EMT) is also affected by the expression of DDRs. Some reports have shown that induction of an EMT phenotype results in transcriptional downregulation of DDR1 and that a predominant DDR2 expression has, as a result, an EMT process towards more malignant cells [70]. Migration and invasion properties are also influenced by these receptors, but this regulation seems to be cell type and receptor isoform dependent [68].

## 5. Lysyl Oxidase

Results from the last decade have demonstrated that breast tumorigenesis is accompanied by collagen crosslinking with the consequent ECM stiffening and increased focal adhesions. The crosslinking process is catalyzed by lysyl oxidase (LOX), a copper-dependent amine oxidase that initiates the covalent cross-linking of collagens and elastin to promote their stability and drive malignant progression [71]. In the cellular complexity of a tumor, it has been demonstrated that LOX can be expressed either by stromal or epithelial cells, in the latter case, associated with a malignant phenotype [72]. Using a murine model of breast cancer, it was demonstrated that LOX expression was stimulated by TGF-β derived, in this case, from epithelial origin, which generates a stiffer collagen matrix that stimulates tumoral progression [73].

Pancreatic ductal adenocarcinoma (PDAC) is a human tumor, almost universally lethal, that displays a highly desmoplastic stromal microenvironment [74]. Using a principal component analysis (PCA), authors have identified a LOX/hypoxia signature associated with poor patient survival. Moreover, these authors were able to demonstrate that inhibition of LOX suppressed experimentally-induced metastasis [75]. Interestingly, it has been suggested that LOX displays a specific role in the late stage of metastasis more than in the growth of the primary tumor [74].

The LOX inhibitor β-aminopropionitrile (BAPN) has been proposed as a therapeutic tool for the inhibition of desmoplasia. Interestingly, this compound is concentrated in some variety of peas (lathyrus pea), and their consumption has been associated with the development of lathyrism, illness characterized by a defective mesenchymal tissue [76]. BAPN has been used in the treatment of keloids and hypertrophic scarring but its use in cancer has not progressed significantly, and the current effort of the industry is oriented towards identifying compounds capable of inhibiting the amine oxidase activity [77].

## 6. The Metabolic Side

Metabolic reprogramming, a phenomenon mostly explained by glucose consumption, has been proposed as one of the most distinctive features of cancer progression [78]. The initial approaches regarding the study on the effect of matrix rigidity on tumor metabolism was directed to tumoral cells. In these studies, it was demonstrated that under a highly dense collagen matrix, breast cancer cells show a decreased utilization of glucose through the tricarboxylic acid (TCA) cycle and an enhanced utilization of glutamine as a fuel source to drive the TCA cycle [79]. In the last decades, it has been proposed that the stromal compartment can exert a relevant role in the development of the metabolic reprogramming phenomenon [80]. One of the central concepts in this proposal is that this metabolic modulation can be achieved in a collaborative manner between stromal and epithelial cell populations with a focus on the mechanisms of substrate transport. Lisanti and colleagues proposed that cancer-associated fibroblasts (CAFs) collaborate with tumor cells by supplementing lactate that, in turn, is consumed by cancer cells and oxidized in the mitochondria for energetic purposes [81]. Therefore, in desmoplastic tumors, the enhanced conversion of glucose to lactate generates lactic acid that is released to the extracellular compartment. 

The functional connection between metabolism and fibrosis was supported by studies using metformin, an agonist of AMP-activated protein kinase (AMPK), which, by reducing TGF-β levels and its signaling pathway, decreases collagen deposition and fibrosis [82]. In our laboratory, by using compliant elastic polyacrylamide gels covalently-linked to type I collagen, we observed that increased stiffness promotes lactate production and glucose uptake by mammary fibroblasts. This response was correlated with the expression of the stromal glucose transporter Glut1 and monocarboxylate transporters MCT4. Moreover, mammary stromal cells cultured on rigid matrices generated soluble factors that stimulated epithelial breast migration in a stiffness-dependent manner. Therefore, we propose that glucose reprogramming and ECM dynamics are highly connected phenomena that act in a cooperative manner [83].

## 7. Conclusions

**Figure 1:**
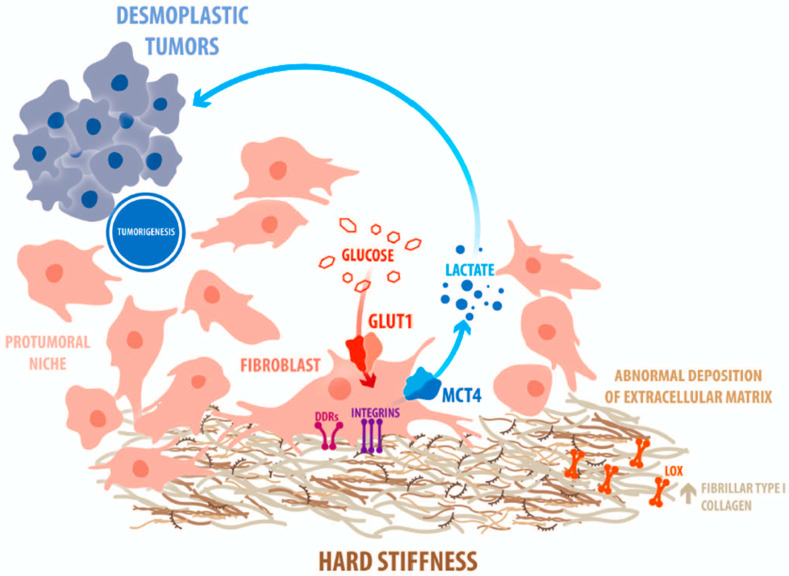
Scheme of a desmoplastic tumor showing a great prevalence of stromal cells that produce and deposit an abundant extracellular matrix. The active metabolic cross-talk between stroma and epithelia is highlighted.

Different experimental approaches have shown that breast cancer is closely linked to modifications in breast stroma. Accumulation of ECM (formed mostly by fibrillar collagen) on the site of tumor development constitutes an active player in tumoral progression. This phenomenon can act in a different temporal way. On one hand, it can promote the establishment of a pro-tumoral niche that predisposes cancer development. On the other hand, it can be structured under the tumor stimulus. These changes not only include the initiation of tumor development, but also the response of cancer to chemotherapeutic approaches. A rigid tumor also perturbs the access of antineoplastic drugs which constitute a strong hurdle to drug-dependent therapeutics. This rather new knowledge underscores the importance of considering the possible microenvironmental effects in the study of tumoral progression and drug development. Figure 1 shows a schematic drawing of the main events involved during extracellular matrix remodeling in desmoplasia.

## Data Availability

The data presented in this study are available on request from the corresponding authors.
